# Optimization of Fermentation Medium for the Production of Glucose Isomerase Using *Streptomyces sp. SB-P1*

**DOI:** 10.1155/2012/874152

**Published:** 2012-07-26

**Authors:** Sheetal Bhasin, H. A. Modi

**Affiliations:** ^1^Department of Biosciences, Maharaja Ranjit Singh College of Professional Sciences, Madhya Pradesh, Indore 452001, India; ^2^Department of Life Sciences, Gujarat University, Gujarat, Ahmedabad 380009, India

## Abstract

The combination of medium ingredients has a profound influence on the metabolic pathways running in the microorganism which regulates the production of numerous metabolites. Glucose isomerase (GI), an enzyme with huge potential in the market, can isomerise glucose into fructose. GI is used widely for the production of High-Fructose Corn Syrup (HFCS). HFCS is used as a sweetener in food and pharmaceutical industries. *Streptomyces* are well-known producers of numerous enzymes including glucose isomerase. An array of 75 isolates was screened for the production of glucose isomerase. The isolate *Streptomyces sp. SB-P1* was found to produce maximum amount of extracellular GI. Sucrose and raffinose among pure carbon sources and corn cob and wheat husk among crude agro residues were found to yield high enzyme titers. Potassium nitrate among pure nitrogen sources and soy residues among crude sources gave maximum production. Quantitative effect of carbon, nitrogen, and inducer on GI was also determined. Plackett-Burman design was used to study the effect of different medium ingredients. Sucrose and xylose as carbon sources and peptone and soy residues as nitrogen sources proved to be beneficial for GI production.

## 1. Introduction

GI (EC 5.3.1.5) catalyses the conversion of glucose into fructose. The most important application of GI is the production of High-Fructose Corn Syrup (HFCS). It is an equilibrium mixture of fructose and glucose. Fructose, also known as fruit sugar, is the sweetest natural sugar and is found in fruits, vegetables and honey. HFCS has wide applications in pharmaceutical and food industries. It is added in medicated syrups, beverages, baking, canning and confectionary items as a sweetening agent [1]. Ge et al. [2] developed a process for continuous production of HFCS by Immobilised Glucose Isomerase (IGI).

GIs characterized from different microbial sources vary in molecular mass from 80 to 195 kDa and are composed of two or four identical subunits. Glucose isomerase from *Streptomyces *is a tetramer composed of four identical polypeptide chains of 43,000 daltons each [1, 3].

Marshall and Kooi in 1957 [4] for the first time reported the production of glucose isomerase from *Pseudomonas hydrophila*. Since then many mesophilic, thermophilic, a few psychrophilic, aerobic, and anaerobic organisms have been reported to produce GI. Streptomycetes have been the organisms of choice for GI production by numerous researchers.

The thrust areas to work upon for medium formulation are independence of xylose and cobalt ions. Xylose works as an inducer for GI in majority of the cases excluding a few like *Actinoplanes missouriensis *[5]. Xylose-independent GI producer or an agro-residue rich in xylose which can substitute for pure and expensive xylose can be useful in making the production technology economic. There are reports on *Bacillus* being used for GI production on xylan containing media also [6]. Most of the researchers have suggested the requirement of cobalt and magnesium in the production medium as cobalt provides thermostability and magnesium is required for optimum activity of the enzyme. The deleterious effects of cobalt on human health forces the elimination of cobalt from production medium [7–9].

Employing a suitable production medium for fermentative production of GI is a very crucial requirement of present time in order to develop an economically viable technology. India being an agrobased country is a rich producer of agroresidues. These residues can be utilized for microbial production processes.

## 2. Materials and Methods

A collection of 75 actinomycetal isolates was developed from compost pit samples. The isolates exhibited cultural and morphological diversity [10]. All the isolates were screened for GI activity and the highest producer of the enzyme *Streptomyces sp. SB-P1* was selected for medium optimisation studies for industrial purpose [11].

### 2.1. Selection of Medium Combination

 GI production is known to be enhanced by the presence of its inducer xylose. The production is also influenced by the presence of magnesium and cobalt ions in some cases. Investigators have used various medium combinations for GI production according to the microorganism employed. The selected isolate *Streptomyces sp. SB-P1* was grown in 11 different media combinations to screen the best combination supporting maximum GI production and biomass accumulation. The medium combinations were Medium No. 1, Bennett's Broth [12]; Medium no. 2, Modified Bennett's Broth [12]; Medium No. 3, Manhas Medium [13]; Medium No. 4, Pawar Medium [14]; Medium No. 5, Kasumi Medium [15]; Medium No. 6, Chen Medium [16]; Medium No. 7, Lobanok Medium [17]; Medium No. 8, Strandberg Medium [18]; Medium No. 9, Srih-Belghith Medium [19]; Medium No. 10, Hasal Medium [6]; Medium No. 11, Dhungel Medium [20]. The flasks containing 20 mL of medium were incubated at 30°C for 96 h at 120 RPM. The centrifuged supernatant of fermented broth was used as crude enzyme extract for determination of GI activity by assay method described by Chen et al. [16]. The reaction mixture for GI assay contained 500 *μ*L of 0.2 M sodium phosphate buffer, 200 *μ*L of 1 M glucose, 100 *μ*L of 0.1 M magnesium sulphate, 100 *μ*L of 0.01 M cobalt chloride, and 200 *μ*L of crude enzyme extract. The final volume of assay mixture was made up to 2 mL. This reaction mixture was incubated in water bath at 70°C for 60 minutes. The reaction was stopped by adding 2 mL of 0.5 M perchloric acid. To 0.05 mL aliquot of above 0.95 mL of distilled water was added. This is followed by the addition of 200 *μ*L of 1.5% cysteine hydrochloride, 6 mL of 70% sulphuric acid, and 200 *μ*L of 0.12% alcoholic carbazole. The intensity of purple colour so developed was estimated spectrophotometrically at 560 nm [21]. One unit of glucose isomerase activity was defined as the amount of enzyme that produced 1 *μ*mol of fructose per minute under the assay conditions described.

The growth response on different medium combinations was measured by estimating the dry weight. The biomass was separated from the nutrient medium by centrifugation and washed with distilled water. The biomass was transferred on a filter paper and kept for drying at 55°C. The dry weight was determined after 24 h.

### 2.2. Screening of Carbon and Nitrogen Sources

 The highest GI production and highest biomass accumulation were achieved on different medium combinations. These two media were used to screen pure and crude sources of carbon and nitrogen. The quantitative effect of best carbon, nitrogen source, and inducer (xylose) was also studied.

### 2.3. Plackett-Burman Design for Screening Medium Components

A set of 14 medium components were screened by Plackett Burman design. The ingredients studied by Placket Burman design were carbon sources (glucose, sucrose, wheat husk, orange bagasse, peanut shell), nitrogen sources (tryptone, peptone, yeast extract, soy flour, potassium nitrate), minerals (magnesium sulphate, cobalt chloride), inducer (xylose), and a buffering agent (di-potassium hydrogen phosphate). The high and low concentrations of medium ingredients used are given in Table 1 and design of experiment is given in Table 2.

## 3. Result and Discussions

### 3.1. Selection of Medium Combination

**Figure 1:**
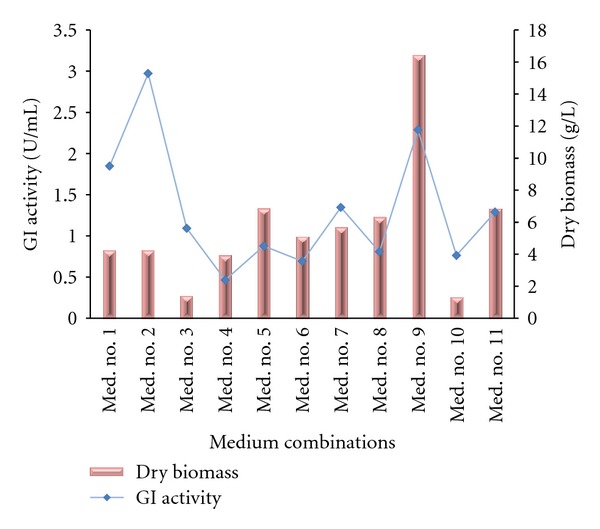
Effect of different media combinations on GI production and dry biomass.

Various medium combinations were tried for the production of GI. Medium used by Srih-Belghith and Bejar [19] were found to give maximum growth and modified Bennett's broth gave maximum enzyme yield. The medium combinations containing xylose were found to produce good amount of enzyme as compared to the media without xylose. Media containing xylose as sole source of carbon exhibited poor growth but high enzyme yield. As also reported by Hasal et al. [7] that glucose and xylose together decreased the biomass yield we found same results in Medium No. 2 where enzyme production was high but growth was less. The highest enzyme production was observed in Medium No. 2 and next was Medium No. 9 which also yielded highest biomass. High biomass production may be due to the high protein content (yeast extract) in the medium as also reported by Givry and Duchiron [22]. The enzyme activity we observed for our isolate *Streptomyces sp. SB-P1 *(1.3 U/mL of fermented broth) was higher than reported by Lobanok et al. [17] (0.14 to 0.73 U/mL) in the production medium used by them. Similar case was observed in Medium No. 3 where our isolate yielded 1.09 U/mL but Manhas and Bala [13] observed a maximum of 0.13 U/mL for their Streptomycete isolates in the same medium. The results of above comparison are graphically represented in Figure 1. As the production of enzyme is associated with the organism's growth, we studied the effect of different carbon and nitrogen sources on both media (one which exhibited maximum GI production and another which was at second position for enzyme production but was best in biomass accumulation).

### 3.2. Screening of Carbon Sources

**Figure 2:**
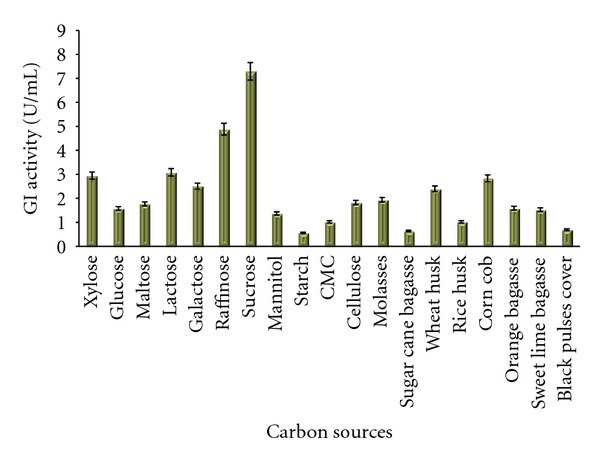
Effect of different carbon sources on the production of GI in Medium No. 1.

**Figure 3:**
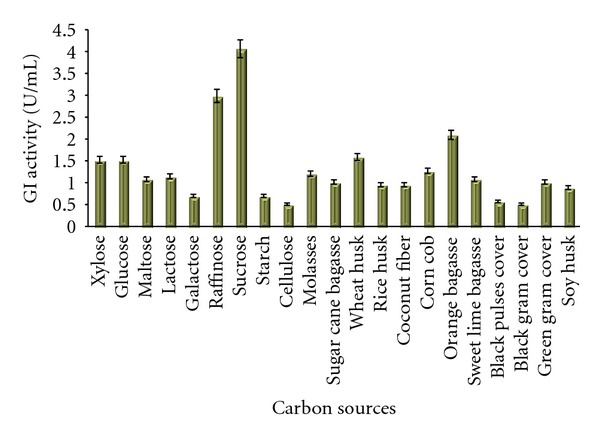
Effect of different carbon sources on the production of GI in Medium No. 9.

Sucrose gave maximum enzymatic yield in both media (Medium No. 2 and 9) followed by raffinose. Among pure carbon sources enzyme yield in flasks containing xylose and lactose were comparable. The results on Bennett's broth base are presented in Figure 2. The results of the effect of carbon sources checked in Medium No. 9 are presented in Figure 3. Lactose also gave better enzyme yield which opens a way to use dairy industry wastes especially whey as a prospective crude and inexpensive carbon source. Hasal et al. [7] studied the effect of various carbon sources to be used along with xylose in order to reduce the amount of expensive xylose in the production medium. They found all the sources except lactose suppressed the yield of GI. Lactose is also reported as an inducer for the enzyme produced by a recombinant *E. coli* containing xylose isomerase gene from *Arthrobacter nicotianae* by Kovalenko et al. [23]. Among the crude sources wheat husk and corn cob yielded high enzyme titres in Medium No. 1 and wheat husk and orange bagasse were good in Medium No. 9. Wheat husk is found to be common in both cases which contain good amount of xylose and xylan. Earlier investigators [13, 17, 24] have also reported high enzyme yield and good growth response of *Streptomyces* in presence of wheat bran. Straw hemicellulose also works as an inducer that can replace xylose and increase the GI yield [16]. Hemicelluloses containing substance is useful as a medium ingredient because of its low cost than xylan and xylose. Alkali-treated hemicelluloses are also used by Chen and Anderson, [25] to produce glucose isomerase.

### 3.3. Screening of Nitrogen Sources

**Figure 4:**
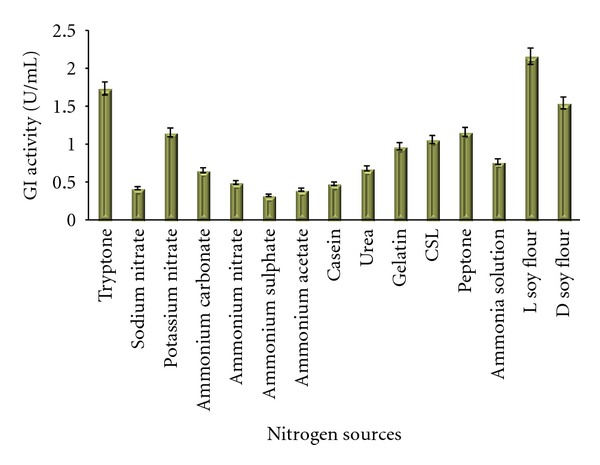
Effect of different nitrogen sources on the production of GI in Medium No. 1.

**Figure 5:**
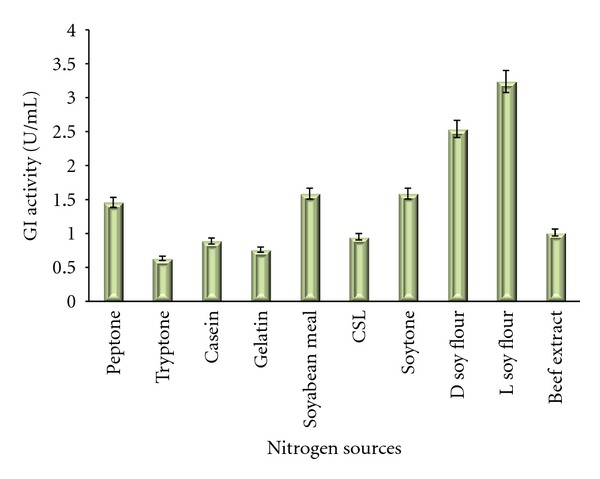
Effect of different nitrogen sources on the production of GI in Medium No. 9.

Various inorganic and organic nitrogen sources were tested for glucose isomerase production. Among the pure nitrogen sources potassium nitrate was found to be more suitable for high yield. There are reports suggesting the inability of inorganic salts in giving higher yields of glucose isomerase but we observed a remarkable increase in the enzyme production in the flasks containing potassium nitrate. Soy flour was pointed out as the best source among the crude components tested. The higher yields with agrobased and industrial residue are a good sign for its usage in industrial production media. Corn steep liquor is reported to increase the enzyme yield by Hasal et al. [7] whereas we did not observe any substantial increase in the enzyme productivity with it as also stated by Deshmukh et al. [26]. There are reports on ammonium salts been promoting GI production but soy residues are less preferred [1]. Givry and Duchiron [22] found *Lactobacillus bifermentans* could produce high amount of glucose isomerase only in presence of organic nitrogen sources like peptone, tryptone or yeast extract. This is the first report to our knowledge stating high production of GI in presence of soy residues. This is again a beneficial result in the context that Madhya Pradesh, India is one of the major producers of soybean and its products so lots of soy residue are also generated. The results on Bennett's broth base are presented in Figure 4. The results of the effect of nitrogen sources checked in Medium No. 9 are presented in Figure 5.

### 3.4. Quantitative Effect of Best Carbon Source: Sucrose

**Figure 6:**
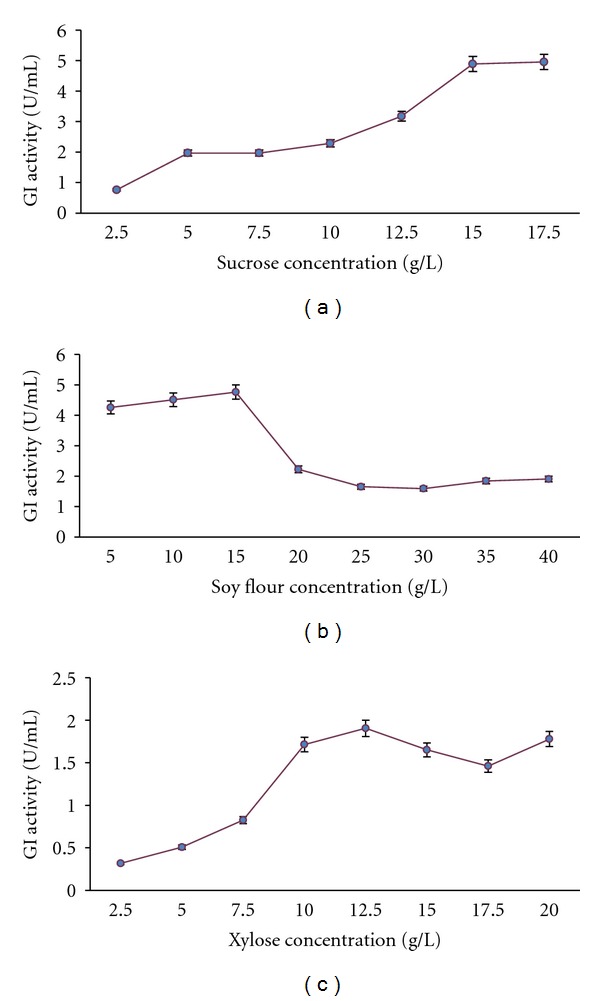
(a) Effect of varying concentration of carbon source on GI production. (b) Effect of varying concentration of nitrogen source soy flour on GI production. (c) Effect of varying concentration of xylose on GI production.

Increasing concentrations of sucrose also increased the enzyme productivity but till a certain limit beyond which there was no substantial increase. There was an increase in the productivity of glucose isomerase from 0.762 U/mL in 2.5 g/L of sucrose to 4.88 U/mL of enzyme in the fermented broth containing 15 g/L of sucrose concentration but further increase did not affect the production. This must be due to the highest sucrose concentration reached which the organism can bear. Sucrose is reported to give high yields of glucose isomerase by earlier researchers also. The results of quantitative effect of sucrose are shown in Figure 6(a).

### 3.5. Quantitative Effect of Best Nitrogen Source: Soy Flour

Production of enzyme increased from 4.2 U/mL to 4.7 U/mL in the medium with increase in soy flour concentration from 5 g/L to 15 g/L but further increase substantially decreased the production. The optimum concentration of 15 g/L is far less as compared to 2.5% corn steep liquor being used by Chen et al. [16]. The literature survey shows that nitrogen content in the media used for the production of GI ranges from 0.3% to 3% and our results are also falling in the same range [13, 20]. The results are shown in Figure 6(b).

### 3.6. Quantitative Effect of Inducer Xylose

The quantitative effect of xylose was studied on glucose isomerase production because it is known as an inducer of the enzyme. Although xylose was not found to give better enzyme yields in carbon source screening but its presence in Medium No. 2 and other media definitely can be related to high enzyme titres. The increase in enzyme production in the fermentation medium was observed from 2.5 g/L of xylose to 12.5 g/L but further increase in the concentration was not useful. Most of the researchers have noticed 1% concentration of xylose serving as good inducer which is near about our result [7, 13, 19]. Givry and Duchiron [22] also observed increase in the enzyme productivity till 1% xylose content. There have been trials for substituting 25 to 75% of xylose with other carbon sources. There have been trials for substitution of xylose by glycerol and sorbitol. Givry and Duchiron [22] observed that arabinose as carbon source yielded higher amount of enzyme than xylose while using *Lactobacillus bifermentans *for the combined production of arabinose isomerase and xylose isomerase. They also proposed the possibility of substituting starch, glucose, sorbitol or glycerol for 75% of xylose. Glucose produces high biomass but lower enzyme yield as compared to pentoses. Paik and Dewey [27] reported the use of xylan in place of xylose as an inducer. The results are presented in Figure 6(c).

### 3.7. Plackett Burman Design for Screening Medium Components

**Figure 7:**
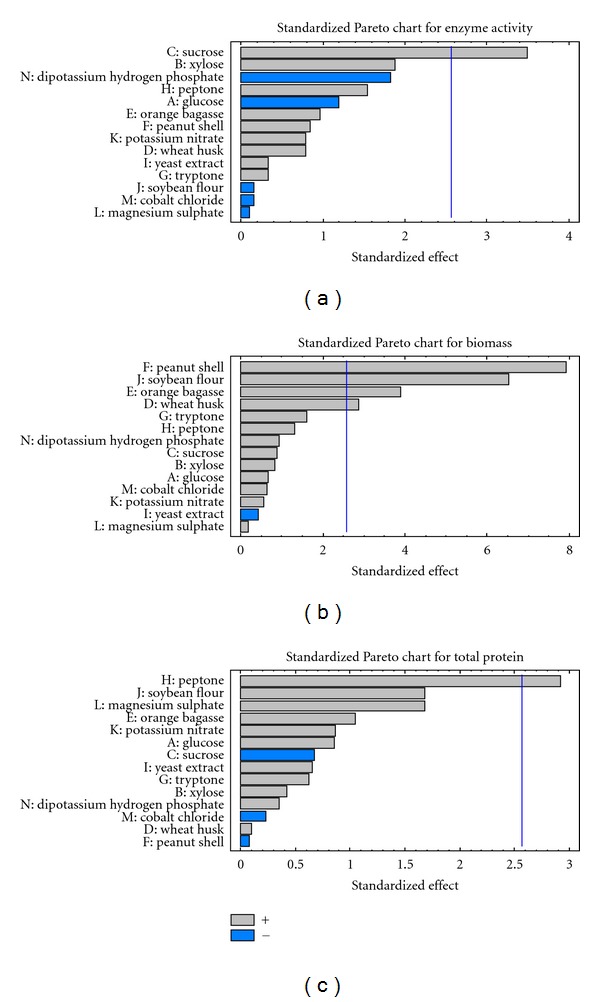
(a) Standardized Pareto chart for GI activity. (b) Standardized Pareto chart for biomass production. (c) Standardized Pareto chart for total protein.

**Figure 8:**
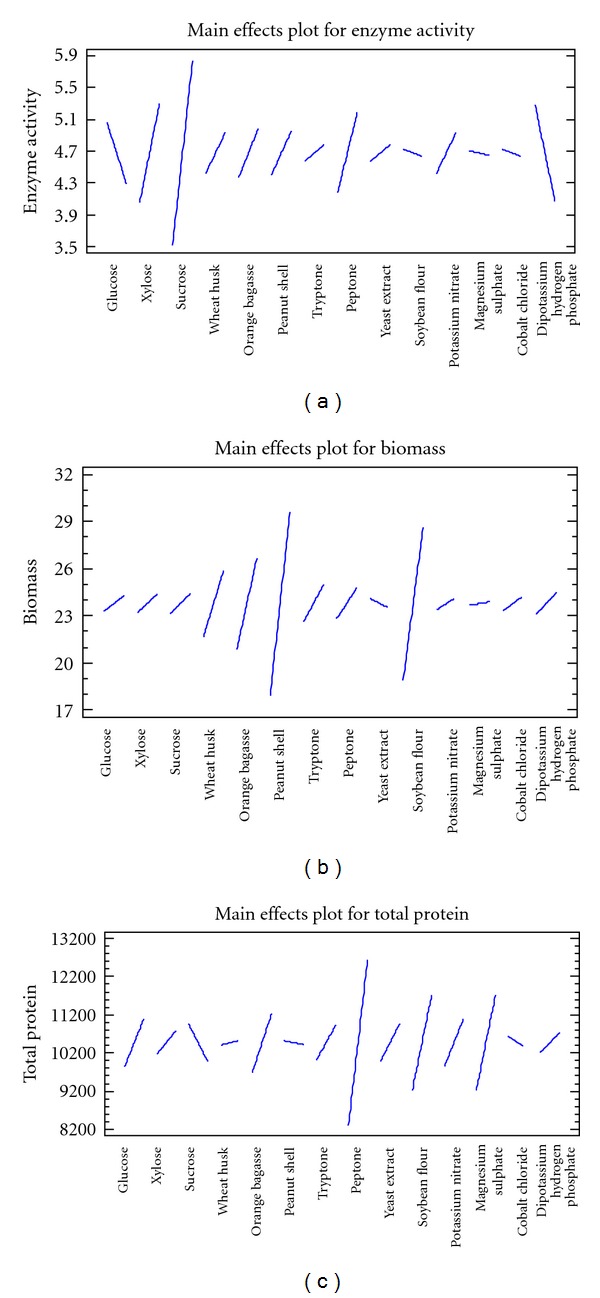
(a) Main effects plot for GI activity. (b) Main effects plot for biomass production. (c) Main effects plot for total protein.

Plackett-Burman's design was used to check the effect of 14 components on glucose isomerase production. The preliminary screening experiments revealed probable sources of carbon and nitrogen which can enhance GI production. Sucrose, wheat bran, and orange bagasse are helpful in enzyme production as carbon sources and soy flour and potassium nitrate as nitrogen source. Other components were selected on the basis of commonly used medium ingredients reported by earlier researchers for glucose isomerase production. The response of the factors was studied in the form of 3 variables, enzyme activity, total protein content, and biomass. Sucrose, xylose, peptone, orange bagasse, peanut shell, potassium nitrate, wheat husk, yeast extract, and tryptone exhibited positive effects on the production of glucose isomerase. This indicates that the enzyme production was enhanced by adding higher concentration of these ingredients. Glucose, soy flour, di-potassium hydrogen phosphate, cobalt chloride, and magnesium sulphate had negative effects which also means that increasing the concentration of these ingredients decreases the production of enzymes. The results are depicted in Table 3. The Pareto chart and main effects plot for enzyme activity response are presented in Figures 7(a) and 8(a), respectively.

Peanut shell, soy flour, orange bagasse, and wheat husk had significant positive effect on biomass yield. Peanut shell hydrolysate is used by researchers in fuel production applications as a good source of sugar [28]. All other ingredients besides yeast extract were found to increase the biomass yield. Total protein content in the fermented broth was also positively influenced by all the medium ingredients other than sucrose, cobalt chloride and peanut shell. The Pareto chart and main effects plot for biomass yield and total protein content response are presented in Figures 7(b), 8(b), 7(c), and 8(c) respectively.

On considering the overall response for production sucrose can be marked as a useful ingredient as it has a positive effect on the enzyme as well as biomass yield. The results indicate wheat husk and orange bagasse are beneficial for the process because they had a positive effect on the entire variables tested that is, enzyme, biomass, and total protein yield. Wheat products have proved to be beneficial in several reports for GI production [13, 19]. As the enzyme we are testing is extracellular so total protein increase also indicates enzyme increase. Among the nitrogenous sources tested peptone exhibited a prominent positive effect in all the three responses therefore this is the next component which can be picked up for further optimization studies. Presence of inorganic nitrogen source does not support biomass as well as enzyme production but organic sources such as peptone, tryptone, and yeast extract are reported to increase GI by many researchers [7, 16, 22, 26]. The presence of potassium nitrate was found to enhance enzyme yield which is different from the above reports.

Magnesium ions exhibited positive effect on the biomass and total protein content but negative effect on GI production whereas all the above researchers have found it's presence beneficial. Presence of cobalt was found to enhance the production marginally in some cases but it was inhibitory for our isolate as also stated by Chen et al. [16].

Epting et al., [29] studied the influence of presence of magnesium, manganese and cobalt on denaturing of the isomerases from different sources. They reported that apoenzyme melted at a lower temperature than the same enzyme containing any of the three metals.

Presence of magnesium is preferred by *Streptomyces sp*. whereas Givry and Duchiron [22] reported the addition of manganese in the fermentation medium resulted in increased production of GI by *Lactobacillus bifermentans*. Presence of other ions (Mg^2+^, Co^2+^, Ca^2+^ and Zn^2+^) also resulted in increased activity, although their effect was less pronounced than that of manganese. Moreover, in the presence of  Co^2+^, Ca^2+^ and Zn^2+^ ions, the yield was less than in the presence of manganese. Other ions (Cu^2+^ and Fe^2+^) inhibited the GI production. Chen et al. [16] also observed lowered GI production in presence of cobalt but enhanced production in presence of iron. Presence of ZnSO_4_, FeSO_4_ and K_2_HPO_4_  besides MgSO_4 _ in the production medium gives better yields [30].

 Presence of di-potassium hydrogen phosphate did not show a positive effect as also noted by Givry and Duchiron [22]. They reported that increased concentration of the phosphate source had no effect or resulted in a very slight reduction in activity. On the other hand, the decrease in phosphate concentration appreciably improved the enzyme production. However, no effect on cell growth was observed.

## 4. Conclusions

 The isolate *Streptomyces sp. SB-P1* was able to produce glucose isomerase on a wide range of carbon and nitrogen sources. The best was sucrose among the pure carbon sources and wheat husk and orange baggase among crude sources. Presence of xylose definitely increased the yield but did not exhibit a significant effect. The important factor for consideration is that presence of cobalt and magnesium salts did not have a significant effect on enzyme production. This helps us in achieving our goal of designing a medium without cobalt ions as it has deleterious effects on health. Presence of peptone exhibited the enhancement in GI activity, biomass, and total protein. The scaling up of process requires transition of medium ingredients to crude sources. The crude sources like wheat husk and orange bagasse gave good yields which is again beneficial for industrial purposes. The concentration of soy flour required for high production is also quiet reasonable which will help in maintaining the economy of the industrial process.

The ingredients we found to be beneficial are sucrose in 1.5% concentration, soy flour in 1.5% concentration, and xylose in 1% concentration. The results indicate that wheat husk and peanut shell can be used as economically available agro-residues in 1% and 1.5% concentrations, respectively, for industrial production process. Use of agro-residue for industrial production of metabolites not only reduces the production cost but also solves the problem of disposal of tonnes of crop residues produced every year in an agriculture-dominated country like India.

## Figures and Tables

**Table 1:** High and low concentrations of 14 ingredients used in Plackett Burman Design for optimization of fermentation medium for GI production.

Ingredients		Glucose	Xylose	Sucrose	Wheat husk	Orange bagasse	Peanut shell	Tryptone	Peptone	Yeast Extract	Soybean flour	Potassium nitrate	Magnesium sulphate	Cobalt chloride	Dipotassium hydrogen phosphate
Concentration (g/L)	High	10.0	10.0	10.0	0	10.0	15.0	10.0	2.0	7.0	20.0	2.5	0.2	0.24	2.0
	Low	1.0	1.0	0	1.0	0	1.0	1.0	1.0	1.0	1.0	0.5	1.0	0.024	0.2

**Table 2:** Plackett Burman design for optimization of fermentation medium for GI production.

Block	Glucose	Xylose	Sucrose	Wheat husk	Orange bagasse	Peanut shell	Tryptone	Peptone	Yeast extract	Soybean flour	Potassium nitrate	Magnesium sulphate	Cobalt chloride	Dipotassium hydrogen phosphate
1	1.0	10.0	10.0	1.0	10.0	15.0	1.0	1.0	1.0	1.0	2.5	0.2	0.24	0.2
1	10.0	10.0	10.0	10.0	00	1.0	10.0	20.0	1.0	20.0	2.5	0.2	0.024	0.2
1	10.0	1.0	10.0	10.0	10	15.0	1.0	1.0	7.0	20.0	0.5	1.0	0.24	0.2
1	1.0	1.0	10.0	10.0	00	15.0	10.0	1.0	1.0	1.0	0.5	1.0	0.024	2.0
1	10.0	1.0	0.0	10.0	10	1.0	10.0	20.0	1.0	1.0	0.5	0.2	0.24	0.2
1	1.0	1.0	10.0	1.0	10	1.0	10.0	20.0	7.0	20.0	0.5	0.2	0.24	2.0
1	10.0	10.0	0.0	1.0	10	15.0	1.0	20.0	7.0	1.0	0.5	0.2	0.024	2.0
1	1.0	10.0	0.0	10.0	10	15.0	10.0	1.0	1.0	20.0	2.5	0.2	0.24	2.0
1	10.0	1.0	10.0	10.0	00	1.0	1.0	1.0	7.0	1.0	2.5	0.2	0.24	2.0
1	1.0	1.0	0.0	1.0	0.0	1.0	1.0	1.0	1.0	1.0	0.5	0.2	0.024	0.2
1	1.0	10.0	10.0	10.0	10	1.0	1.0	20.0	7.0	1.0	2.5	1.0	0.024	0.2
1	10.0	10.0	0.0	1.0	0.0	1.0	10.0	1.0	7.0	1.0	2.5	1.0	0.24	2.0
1	10.0	1.0	0.0	1.0	0.0	15.0	1.0	20.0	1.0	20.0	2.5	1.0	0.24	0.2
1	10.0	10.0	0.0	10.0	10	1.0	1.0	1.0	1.0	20.0	0.5	1.0	0.024	2.0
1	10.0	1.0	10.0	1.0	10	15.0	10.0	20.0	1.0	1.0	2.5	1.0	0.024	2.0
1	1.0	10.0	10.0	1.0	0.0	1.0	1.0	20.0	1.0	20.0	0.5	1.0	0.24	2.0
1	1.0	10.0	0.0	10.0	0.0	15.0	10.0	20.0	7.0	1.0	0.5	1.0	0.24	0.2
1	1.0	1.0	0.0	1.0	10	1.0	10.0	1.0	7.0	20.0	2.5	1.0	0.024	0.2
1	1.0	1.0	0.0	10.0	0.0	15.0	1.0	20.0	7.0	20.0	2.5	0.2	0.024	2.0
1	10.0	10.0	10.0	1.0	0.0	15.0	10.0	1.0	7.0	20.0	0.5	0.2	0.024	0.2

*Note*. The concentration of ingredients is according to g/L.

**Table 3:** Results for Plackett Burman screening design for GI production.

Run	GI Activity (U/mL)	Biomass (g/L)	Total Protein (g/L)
1	7.62	24.85	4.04
2	7.05	26.09	9.89
3	4.57	36.68	8.37
4	4.19	27.00	5.56
5	3.62	18.35	13.43
6	4.57	28.12	9.96
7	4.19	26.28	11.48
8	4.19	40.67	10.83
9	5.14	10.27	74.36
10	1.52	2.28	12.99
11	7.62	19.31	14.44
12	0.76	11.56	8.88
13	3.05	31.99	14.44
14	2.86	26.41	11.48
15	5.72	26.34	14.44
16	5.91	16.87	14.44
17	6.86	23.07	11.19
18	4.95	19.71	13.93
19	3.24	31.06	12.70
20	5.91	28.71	11.12
